# “Roof” technique—a modified aortotomy closure in Y-incision aortic root enlargement upsizing 3-4 valve sizes

**DOI:** 10.1016/j.xjtc.2022.01.006

**Published:** 2022-01-26

**Authors:** Bo Yang, Aroma Naeem, Sarah Palmer

**Affiliations:** Department of Cardiac Surgery, Michigan Medicine, Ann Arbor, Mich

**Figure:**
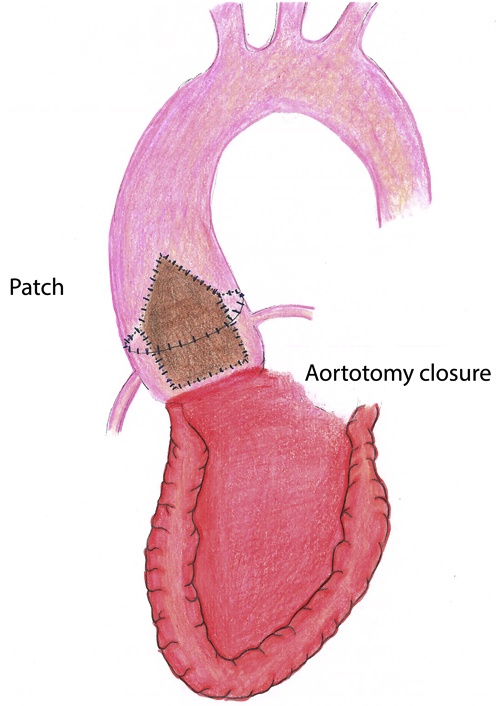
Illustration of “roof” modification of Y-incision/rectangular patch aortotomy closure.

**Central Message**

The “roof” technique of aortotomy closure can enlarge the proximal ascending aorta adequately in Y-incision/rectangular patch root enlargement.

See Commentary on page 37.

We previously described aortic root enlargement with a Y-incision/rectangular patch to enlarge the aortic annulus by 3 to 4 valve sizes with partial aortotomy.^1^, ^2^, ^3^ In patients with a small ascending aorta (<30 mm), the closure of partial aortotomy could be an issue for future transcatheter aortic valve replacement (TAVR), since it may not enlarge the proximal ascending aorta adequately (Figure E1). Here, we describe a modification to the closure of the aortotomy with the “roof technique” to enlarge the sinotubular junction and proximal ascending aorta and to prepare patients for future TAVR (Figure 1).

**Figure 1:**
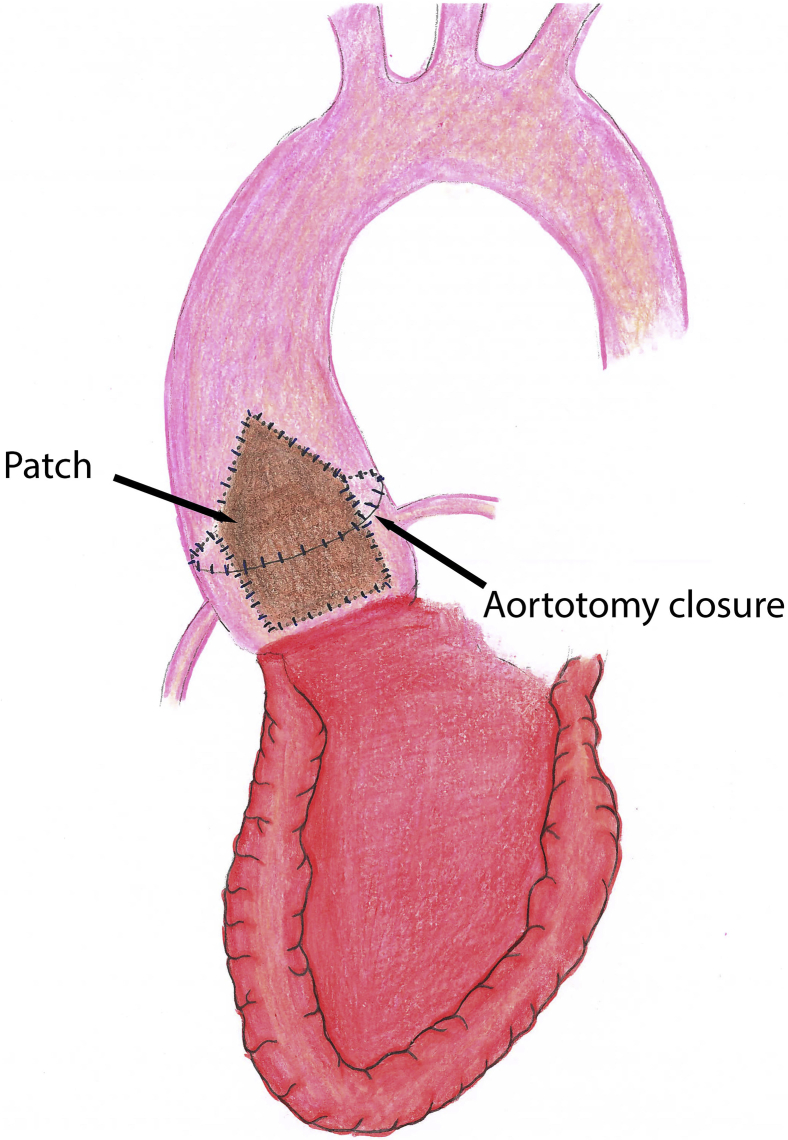
Illustration of the rectangular patch with the triangular shape at the top incorporating into the proximal ascending aorta to enlarge the sinotubular junction and proximal ascending aorta, the suture line sewing the patch to the aortomitral curtain/mitral annulus, aortic annulus after Y-incision through the aorto-mitral curtain, and closure of the aortotomy.

The case was a 62-year-old female patient with body height of 5′1′′ (155 cm), body weight of 85 kg, body mass index of 36 kg/m^2^, and body surface area of 1.8 m^2^. The patient presented with a calcified bicuspid aortic valve with severe aortic stenosis, a mean gradient of 57 mm Hg, and a peak gradient of 95 mm Hg. The annulus size was measured at 19 mm after excision of the leaflets and annular debridement of calcium. After Y-incision/rectangular patch root enlargement, a 25 Magna Ease (Edwards Lifesciences) aortic bioprosthesis was implanted. Postoperative mean gradient was 6 mm Hg across the aortic valve and 3 mm Hg across the left ventricular outflow tract. This patient was discharged without blood transfusion or complications and consented for this case report.

### Details of Aortic Root Enlargement

Details of aortic root enlargement are discussed in Video 1. A complete transverse aortotomy was made 1 cm above the sinotubular junction (STJ). The stenotic aortic valve was excised, and the annulus was debrided. The Y incision was made from the aortotomy, through the left-non commissure post into the aortomitral curtain. The incision was extended in a “Y” fashion undermining the left and noncoronary aortic annulus to their respective nadir, but not reaching the muscular portion on the left or the membranous septum on the right. A rectangular-shaped Hemashield Dacron patch (Maquet, Getinge Group) was trimmed in width slightly longer than the distance between the 2 cusp nadirs. This patch was sewn to the aortomitral curtain/mitral annulus from left to right fibrous trigone with running 4-0 PROLENE suture (Ethicon). The suture line was transitioned to the undermined aortic annulus at the nadir of both the left and noncoronary sinuses, sutured along the longitudinal length of the patch up to the level of the transverse aortotomy incision, and secured. The valve sizer upsized by 3 sizes was placed in the enlarged root touching 3 nadirs of the aortic annulus and the position of the sizer on the patch was marked to guide the placement of valve sutures. The nonpledgeted 2-0 ETHIBOND sutures (Ethicon) were placed along the native aortic annulus in a noneverting fashion and from outside in on the patch. The bioprosthesis was placed with one strut facing the left–right commissural post to ensure the left and right coronary ostia were at the sides of this strut without occlusion. The sutures at the nadirs of the noncoronary and the left coronary sinuses, which were the lowest points of the aortic annulus, were tied first to seat the valve well and to prevent paravalvular leak. The patch above the bioprosthesis was then trimmed in a triangular shape like a roof, and an additional 2 cm longitudinal aortotomy was made at the posterior side of the ascending aorta to match the 2 sides of the triangle-shaped patch (the roof). The aortotomy was then closed with the triangle patch inserted into the proximal ascending aorta with 4-0 PROLENE suture (Figure 2). Postoperative computed tomography angiogram (CTA) at 3 months showed the root, STJ, and proximal ascending were all adequately enlarged compared with the preoperative CTA (Figure 2, *E* and *F*).

**Video 1:**
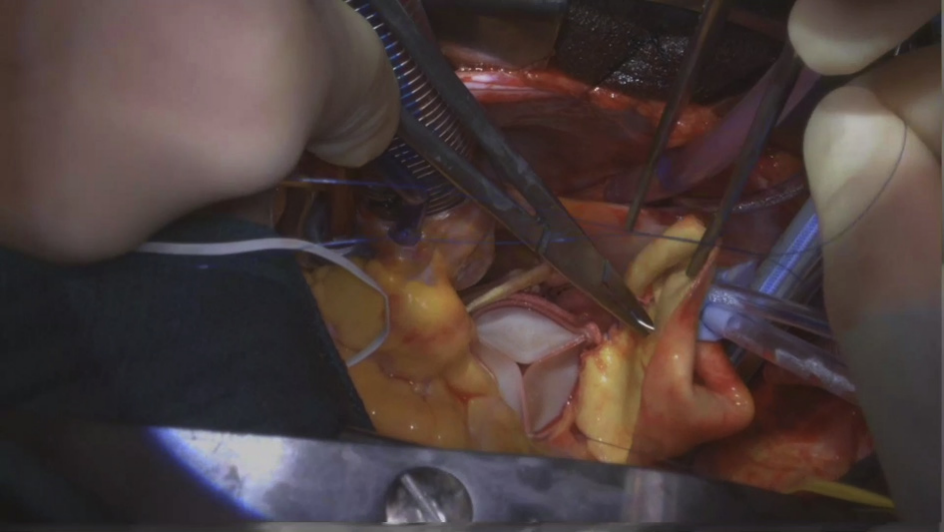
Operative video describing “roof” technique—modification of closure of the aortotomy for “Y” incision to enlarge the aortic annulus by 3 to 4 valve sizes. Video available at: https://www.jtcvs.org/article/S2666-2507(22)00033-5/fulltext.

**Figure 2:**
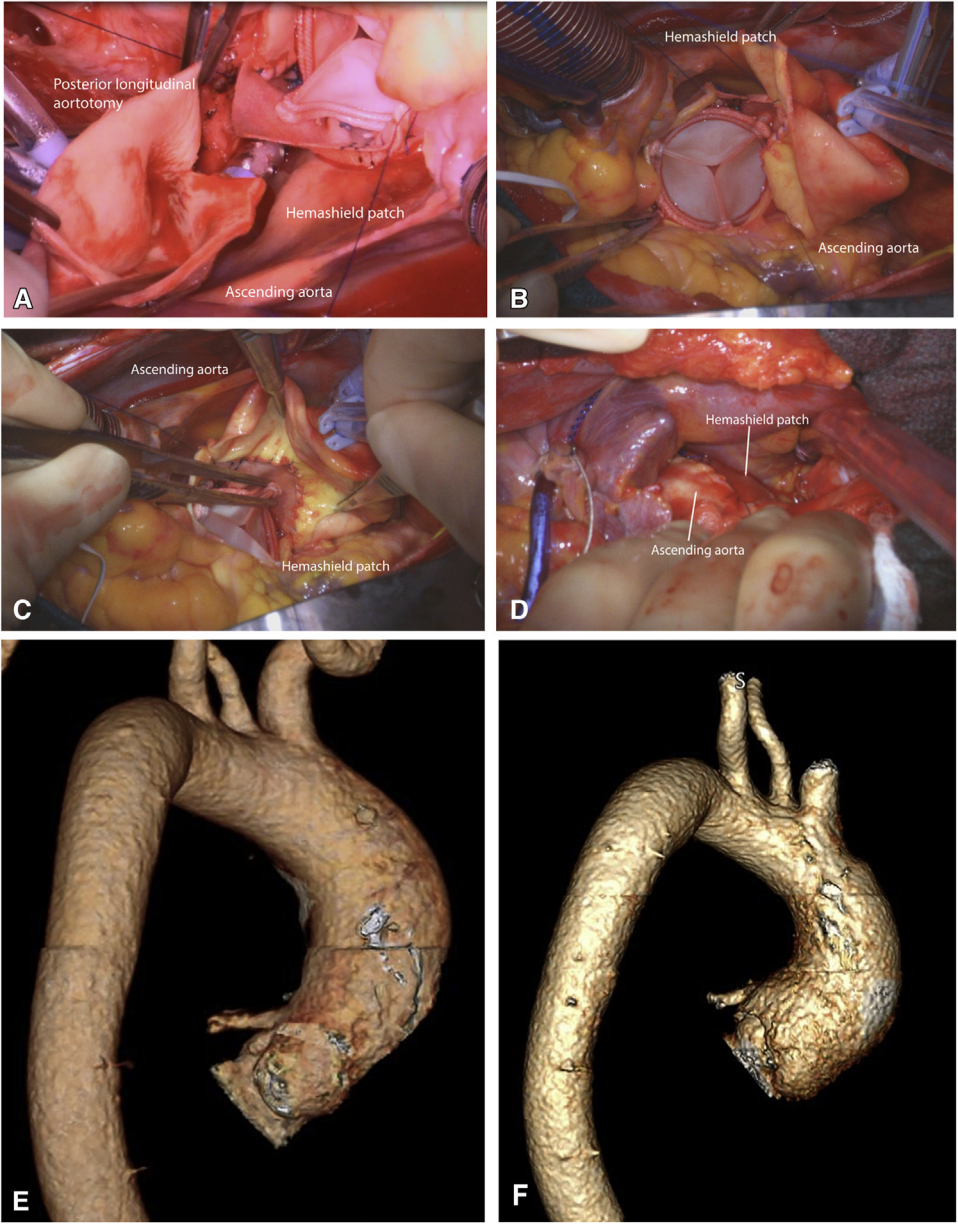
A, The rectangular patch was trimmed into a triangular shape and a longitudinal aortotomy was made to make to incorporate the triangular shape patch into the closure of the complete aortotomy to enlarge the new STJ and proximal ascending aorta. B, The 25 Magna Ease valve (3-valve size upsized); (Edwards Lifesciences) was seated in the enlarged root and triangular patch was partially anastomosed to the longitudinal aortotomy in the ascending aorta. C, The triangular portion (roof) was completely anastomosed to the ascending aorta posteriorly. D, External view of the complete aortotomy closed incorporating the patch at the posterior side of the ascending aorta. E, The preoperative CTA showed the aortic root was 25 × 27 mm, and the STJ was 25 mm. F, The 3-month postoperative CTA showed the aortic root was 35 × 37 mm, and the new STJ was 35 mm. The valve-to-coronary distance was 6 to 7 mm for both coronary ostia.

## Discussion

**Figure E1:**
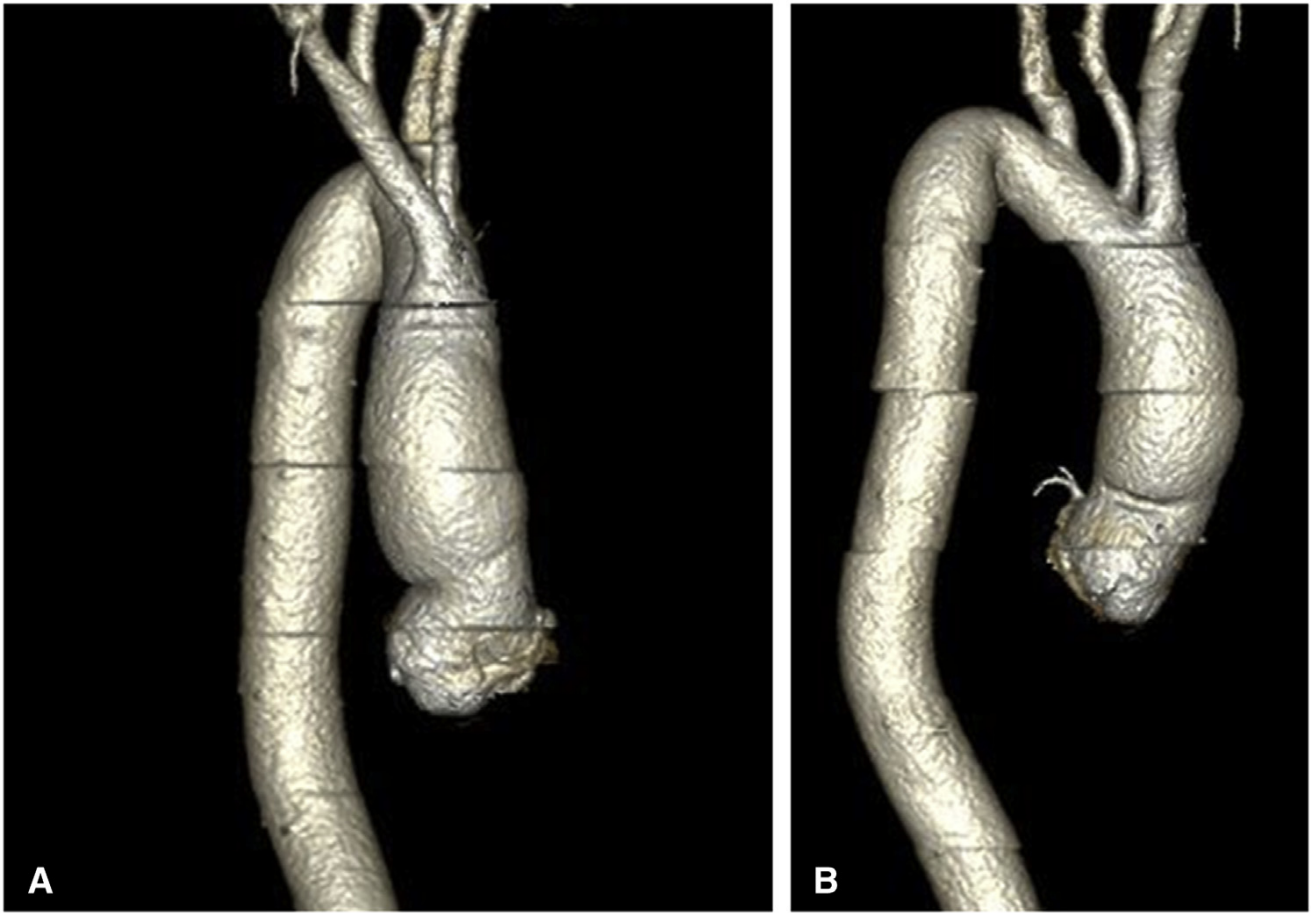
Postoperative computed tomography angiogram at 3 months shows the mild narrowing of the proximal ascending aorta at the closure of partial aortotomy. A, Front view; B, lateral view.

We previously used partial aortotomy for Y-incision/rectangular patch for aortic root enlargement.^1^, ^2^, ^3^ On the follow-up CTA, we found that in some patients with a small aorta (2-2.5 cm), the closure of partial aortotomy did not enlarge the proximal ascending aorta despite the aortic root and SJT were extensively enlarged (Figure E1). By incising the posterior wall longitudinarly about 2 cm, then trimming the rectangular patch above the bioprosthesis into a triangular shape (like a roof), we gradually enlarged the ascending aorta from proximal to distal (Figures 1 and 2). Complete aortotomy also made the visualization and exposure of the root easier for the root enlargement with a Y-incision/rectangular patch. The closure of the aortotomy was easier with roof technique. This approach has become our first choice for Y-incision root enlargement. Attention needs to be paid at the 3 corners of the “roof” (triangular portion of the patch) during closure of the aortotomy for good hemostasis. The roof technique is not needed if the ascending aorta needs to be replaced.

## Conclusions

The modification of the aortotomy closure (roof technique) in Y-incision/rectangular patch root enlargement is simple and effective to enlarge the new STJ and proximal ascending aorta and to prepare patients for future valve-in-valve TAVR.

## Supplementary Data

Video 1 Operative video describing “roof” technique—modification of closure of the aortotomy for “Y” incision to enlarge the aortic annulus by 3 to 4 valve sizes. Video available at: https://www.jtcvs.org/article/S2666-2507(22)00033-5/fulltext.
